# Octocoral dynamics over a decade on Florida’s coral reef

**DOI:** 10.1038/s41598-025-21610-5

**Published:** 2025-10-28

**Authors:** Ronen Liberman, Alexandra Hiley, Lindsay K. Huebner, Michael A. Colella, Rob R. Ruzicka, David S. Gilliam, Nicholas P. Jones

**Affiliations:** 1 National Coral Reef Institute, Nova Southeastern University, 8000 N Ocean Drive, Dania Beach, Florida 33004 USA; 2 Fish & Wildlife Research Institute, Florida Fish & Wildlife Conservation Commission, 100 8th Avenue SE, Saint Petersburg, Florida 33701 USA

**Keywords:** Benthic cover, Density, Hurricanes, Gorgonian, Long-term monitoring, Marine heatwave, Recruitment, Community ecology, Ecological modelling, Population dynamics

## Abstract

In recent decades, studies have documented increased octocoral cover and density on western Atlantic reefs, suggesting their potential to thrive in areas once dominated by scleractinians. On Florida’s Coral Reef (FCR), octocorals are among the most abundant benthic taxa, and octocoral cover has demonstrated resilience, despite frequent acute disturbances that have decimated scleractinian populations. While octocoral cover is an important metric, particularly when assessing broad patterns, it overlooks key demographic processes, such as recruitment and survival, that underpin resilience. This study explored the spatiotemporal trends in arborescent octocorals across FCR using three metrics: percent cover, density, and the recruit density of target species. Using a long-term dataset across FCR from 2013 to 2023, generalized mixed-effects models revealed that temporal variations occurred predominantly at regional habitat scales. Octocoral cover declined slightly in most habitats from 2013 to 2023 (1.67% overall), but density increased overall by 33.7%, indicating a shift in the community composition due to several disturbances during the study period. Recruit density surged in the latter half of the study, leading to an increase in density in some habitats that surpassed pre-disturbance levels. Even though octocoral density was quickly restored by abundant recruitment after these disturbances, successive years of growth and survival were still needed to attain a percent cover equal to pre-disturbance values. Marine heatwaves in 2014 and 2015 had minimal impacts on octocoral cover or density, whereas Hurricane Irma (2017) caused significant region-wide declines in both metrics. The combined effects of Hurricane Ian (2022) and the onset of the 2023 marine heatwave likely drove significant declines in cover and density of octocorals in the Florida Keys and Dry Tortugas at the end of the study period. These findings suggest that some species of octocorals demonstrate a remarkable resilience, with populations recovering rapidly through recruitment following acute disturbances across FCR habitats. This multi-metric approach provides a comprehensive framework for understanding octocoral dynamics and their responses to environmental stressors on FCR.

**Supplementary Information:**

The online version contains supplementary material available at 10.1038/s41598-025-21610-5.

## Introduction

Coral reefs are facing significant pressures from global climate change and local anthropogenic impacts. Long-term observations have revealed significant declines in coral cover in most regions during the last several decades^1–5^. These changes have had severe implications for coral reef community structure and biodiversity^6,7^. Many reefs have experienced pronounced shifts in benthic community composition globally, with losses of scleractinian coral succeeded by increases in non-reef-building organisms such as macroalgae, sponges, and octocorals^8–10^. In the tropical western Atlantic, increased octocoral cover and density have been observed in several locations, including Cuba, Florida Keys, and the U.S. Virgin Islands^11–17^. Furthermore, data across the Caribbean region suggest that the abundance of octocorals increased by 84% between 1968 and 1990 and 2015–2019^15^. In many locations, these increases have occurred following declines in scleractinian cover and have persisted under the conditions that have driven these declines [e.g.,^13,17^]. However, the reasons for octocoral ecological success under these conditions and the implications of these changes on western Atlantic reefs are still poorly understood. There is also a limited understanding of whether reefs that exhibit these changes will persist as alternative stable state or serve as a temporal stepping stone, leading to a novel configuration of benthic reef communities^18,19^.

Like many other reefs in the western Atlantic, Florida’s Coral Reef (FCR) has experienced declines in scleractinian coral cover in recent decades and has been subjected to chronic anthropogenic pressures and acute disturbances^11,16,20,21^. Concomitantly, increased cover of other taxa, including octocorals, sponges, and macroalgae, has been observed throughout the region^11,16^. However, temporal trends in benthic community composition have varied among sub-regions and habitats within the FCR. In some habitats, scleractinian coral cover has been consistently less than 1% since the start of monitoring in 2004^16^. Other habitats had 15–20% cover a decade ago but have continued to experience persistent declines^16^. Spatial variation in community structure is significant between these habitats, but octocorals have been consistently documented as one of the most dominant taxa across FCR habitats^16^. Nevertheless, varying spatiotemporal trends in cover have been reported. In the Florida Keys, an increase in octocoral cover was observed between 1999 and 2009^11^, but from 2004 to 2018, it significantly declined in some locations following two acute disturbances: a severe cold-water event in 2010^22,23^ and Hurricane Irma in 2017^16^. Elsewhere on FCR, both significant increases and decreases in octocoral cover were reported from 2004 to 2018, depending on sub-region and habitat^16^.

Traditionally, changes in percent cover, including both holdfast and canopy cover of arborescent taxa, in addition to the cover of encrusting taxa, have been used to assess trends in octocoral dynamics on FCR^11,16^. However, because most octocoral taxa in our study locations are arborescent, their morphometric traits, such as branch pattern and thickness, can disproportionately inflate their contribution to canopy cover, which then weakens the correlations between cover and abundance metrics^24^. A comparative analysis of both octocoral cover and density is therefore critical for a better understanding of the spatiotemporal changes in the number of octocoral colonies and for estimating changes in their overall estimated size across FCR.

Here, we examine the temporal changes in arborescent octocoral cover and density across multiple regions and habitats between 2013 and 2023. This period is characterized by frequent and severe disturbances, including marine heatwaves, hurricanes, and stony coral tissue loss disease (SCTLD), which strongly impacted FCR’s scleractinian coral assemblages^16,21,25^. Our primary aim was to assess temporal changes in octocoral cover and density in relation to these disturbances to better understand octocoral resistance and resilience. While Jones et al.^16^ reported resilience in octocoral cover during the first portion of this study period (2013–2018), the underlying mechanism for this pattern remains unclear, as changes could occur through growth of remnant adult colonies (i.e., no change in recruit or adult density) and/or recruitment (i.e., increase in recruit and later adult density). We therefore also examined spatiotemporal changes in the density of recruits (individuals ≤ 5 cm high), to elucidate the drivers of octocoral recovery following disturbances. This study provides the most comprehensive assessment of octocoral dynamics across FCR to date, representing one of the largest long-term datasets examining octocoral population changes across multiple spatial scales in the western Atlantic.

## Results

### Model selection and spatial scale of temporal trends

Temporal variation in octocoral cover, density, and target species recruit density occurred at multiple spatial and temporal scales (Table 1). The minimum adequate model for each metric indicated that spatiotemporal changes varied most strongly by regional habitat and explained the largest amount of variance across all metrics. Random effect variances indicated a high amount of variation between sites, with less variation between transects at a site. The generalized linear mixed models (GLMMs) suggested that models that included site depth as well as regional habitat were equally as good as those without (dAIC < 2; Table 1); therefore, we retained the most parsimonious model: year × regional habitat.

**Table 1:** Candidate models for octocoral cover, density, and target species recruit density.

Octocoral metric	Candidate model	dAIC	Marginal R^2^	Conditional R^2^
Cover	Year × sub-region + depth + regional habitat + (1|site/transect)	185.7		
	Year x depth + (1|site/transect)	2492.1		
	Year × region + (1|site/transect)	1880.9		
	Year × region + regional habitat + (1|site/transect)	190.6		
	Year × region + depth + (1|site/transect)	1882.0		
	Year × sub-region + (1|site/transect)	206.2		
	Year × sub-region + regional habitat + (1|site/transect)	190.6		
	Year × sub-region + depth + (1|site/transect)	204.7		
	**Year × regional habitat + (1|site/transect)**		**0.062**	**0.117**
	Year × regional habitat + depth+ (1|site/transect)	0.3	**0.063**	**0.115**
Density	Year × sub-region + depth + regional habitat + (1|site/transect)	48		
	Year x depth + (1|site/transect)	174.8		
	Year × region + (1|site/transect)	114.3		
	Year × region + regional habitat + (1|site/transect)	116.9		
	Year × region + depth + (1|site/transect)	114.6		
	Year × sub-region + (1|site/transect)	42.1		
	Year × sub-region + regional habitat + (1|site/transect)	46.1		
	Year × sub-region + depth + (1|site/transect)	42.2		
	**Year × regional habitat + (1|site/transect)**		**0.265**	**0.9**
	Year × regional habitat + depth + (1|site/transect)	1.8	**0.265**	**0.9**
Number of recruits	Year × sub-region + depth + regional habitat + (1|site/transect)	76.7		
	Year x depth+(1|site/transect)	106.2		
	Year × region + (1|site/transect)	50		
	Year × region + regional habitat + (1|site/transect)	49.9		
	Year × region +depth+ (1|site/transect)	51.5		
	Year × sub-region + (1|site/transect)	47.1		
	Year × sub-region + regional habitat + (1|site/transect)	44.6		
	Year × sub-region +depth+ (1|site/transect)	48.6		
	**Year × regional habitat + (1|site/transect)**		**0.116**	**0.198**
	Year × regional habitat + depth+(1|site/transect)	**1.9**	**0.116**	**0.197**

Fitted model, in bold, chosen based on the candidate model with the lowest Akaike information criterion (AIC); shown are the delta AIC (dAIC) for all tested models. Marginal R2 is calculated using fixed effects only, while the conditional R2 includes fixed and random effects, from fitted models

**Fig. 1:**
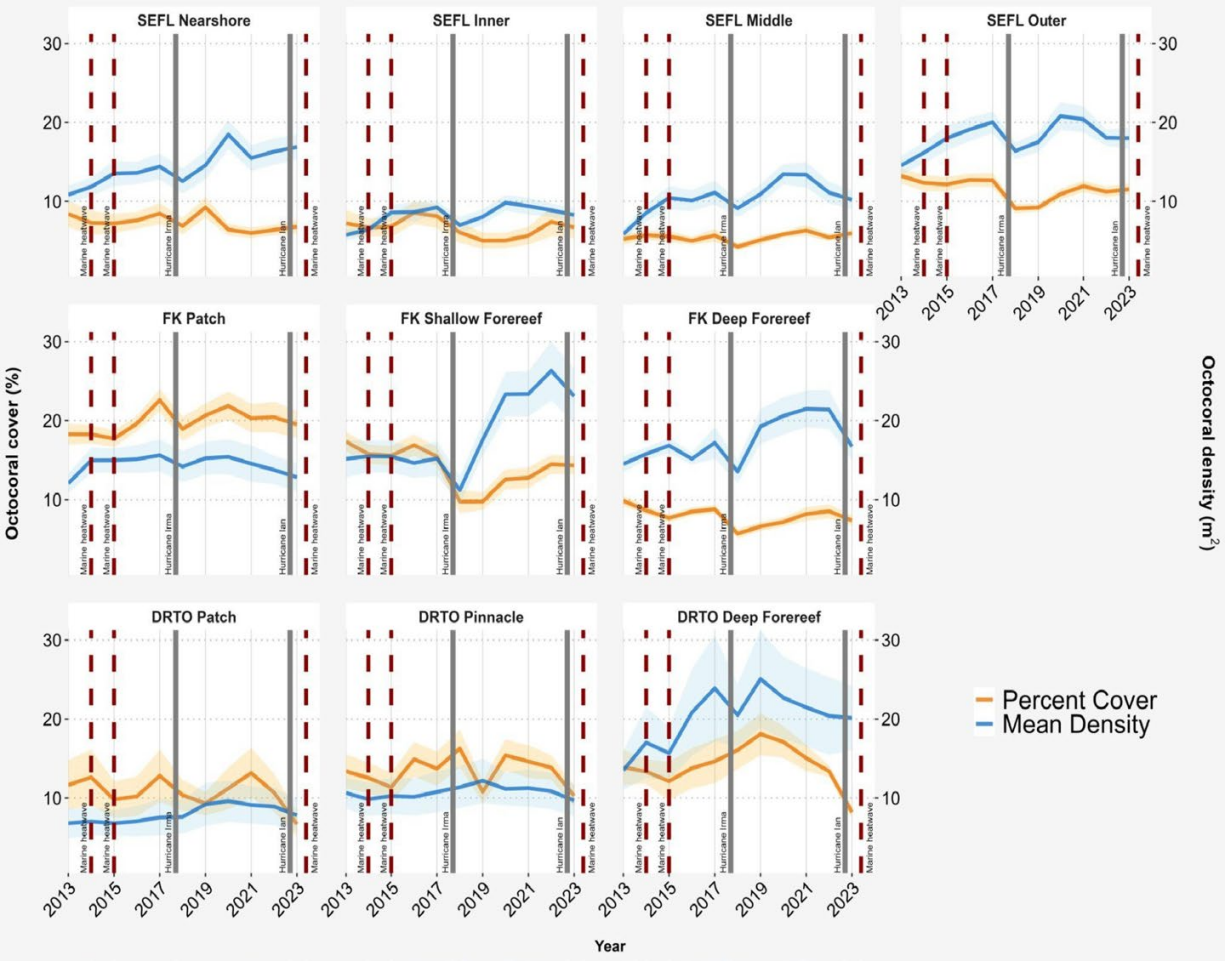
Arborescent octocoral mean (± SE) percent cover (orange) and density (blue) from 2013–2023 in 10 regional habitats on Florida’s Coral Reef. The regional habitats are organized by Southeast Florida (SEFL, top panel), Florida Keys (FK, middle panel), and Dry Tortugas (DRTO, bottom panel). The depth increases left to right in each panel, but comparable depths are found between the SEFL nearshore and inner reefs and between the FK patch and shallow forereefs. Major disturbance events, including marine heatwaves and hurricanes, are indicated using vertical dashed and solid lines, respectively. Interannual significant differences in cover and density can be found in supplementary Figs. S3, S4.

## Regional octocoral temporal changes from 2013 to 2023

**Fig. 2:**
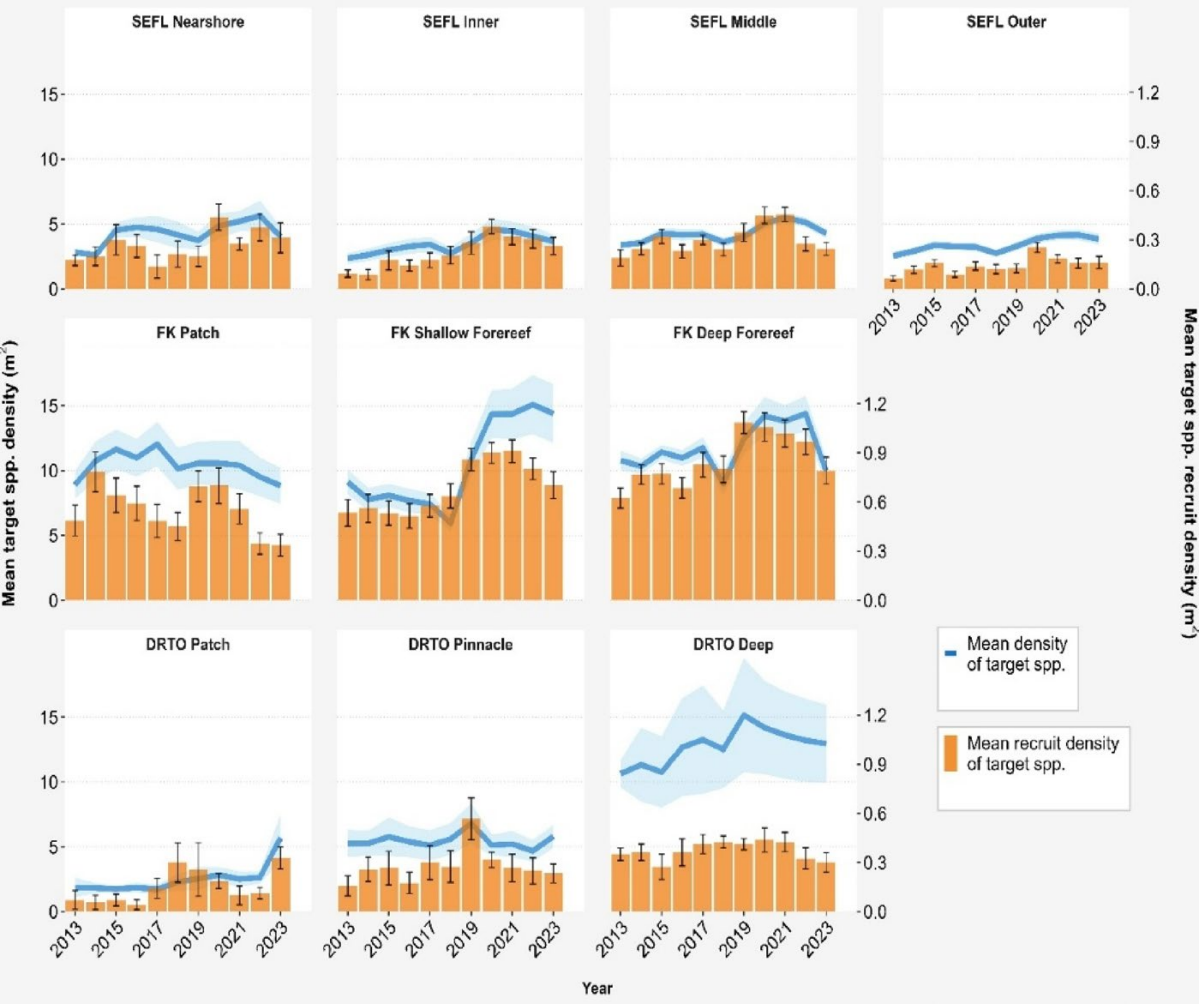
Mean (± SE) density of target octocoral species (blue) and of target species recruit density (≤ 5 cm height; orange) from 2013–2023 in 10 regional habitats on Florida’s Coral Reef. The regional habitats are organized by Southeast Florida (SEFL, top panel), Florida Keys (FK, middle panel), and Dry Tortugas (DRTO, bottom panel). In the FK and DRTO, the target species included *Pseudoplexaura porosa*,* Antillogorgia bipinnata*,* Antillogorgia americana*,* Gorgonia ventalina*, and *Eunicea flexuosa*; only the last three species were targeted in SEFL. The depth increases left to right in each panel, but comparable depths are found between the SEFL nearshore and inner reefs and between the FK patch and shallow forereefs. Major disturbance events, including marine heatwaves and hurricanes, are indicated using vertical dashed and solid lines, respectively. Interannual significant differences in target species recruit density can be viewed in supplementary Fig. S5.

**Fig. 3:**
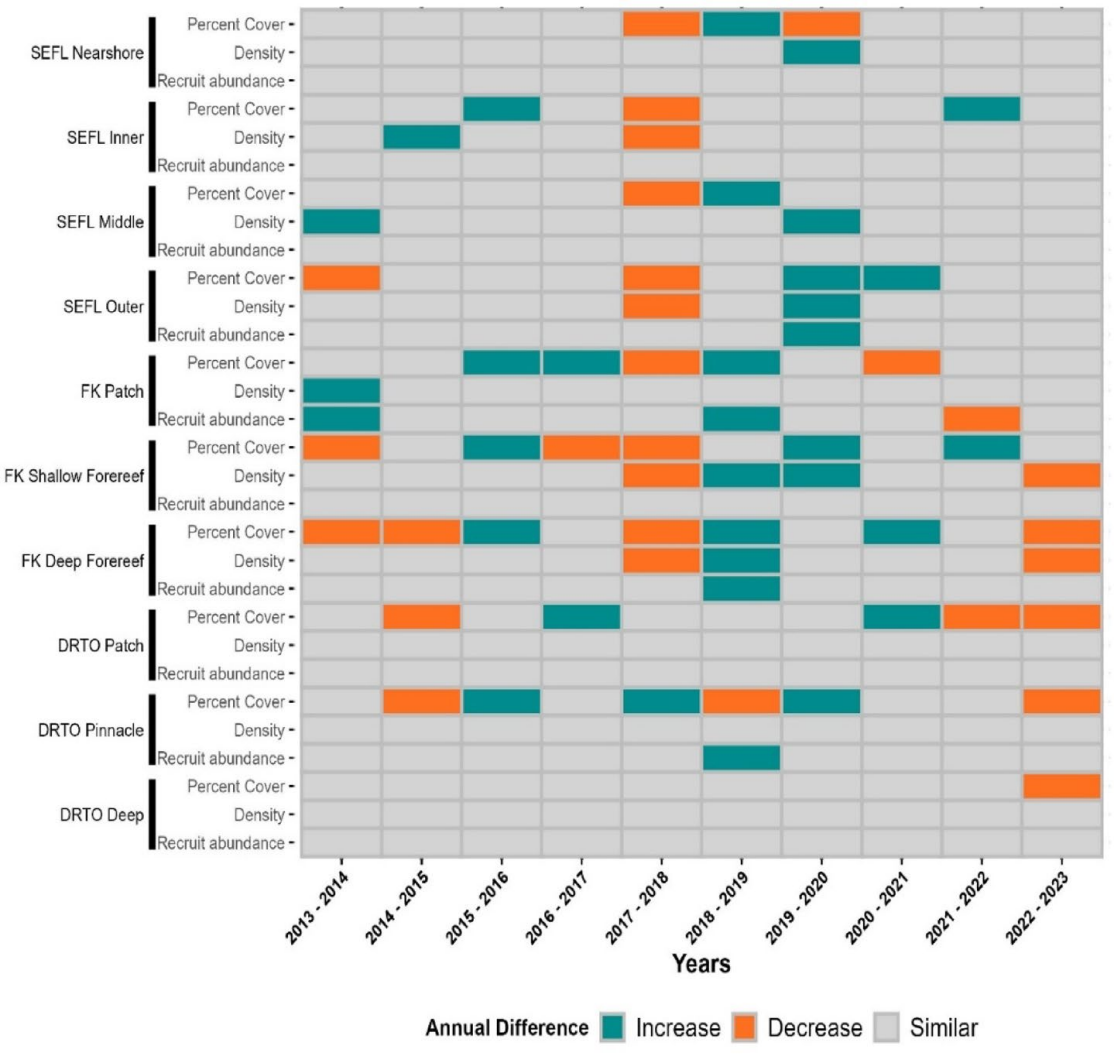
Year-to-year statistically significant increases (teal) or decreases (orange) and non-significant differences (gray) in arborescent octocoral cover, density, and target species recruit density from 2013 to 2023 in 10 regional habitats on Florida’s Coral Reef. Regional habitats are organized by Southeast Florida (SEFL), Florida Keys (FK), and Dry Tortugas (DRTO). Year-to-year comparisons identified by emmeans post-hoc comparisons of fitted generalized linear mixed models (GLMMs, *p* < 0.05); comparisons with similar values are indicated in grey.

GLMM analysis revealed significant but contrasting patterns of spatiotemporal variation in octocoral cover, density, and target species recruit density from 2013 to 2023 within the three regions of the FCR (Figs. 1, S1–S3; Table S1). Despite interannual fluctuations, octocoral cover significantly decreased from 2013 to 2023 in 6 out of 10 regional habitats, spanning all three regions (emmeans comparisons, *p* < 0.01; Figs. 1 and 3; Table S1). Octocoral cover decreased in 3 additional habitats, while only Florida Keys patch reefs showed a slight increase during the study period (*p* > 0.05, Table S1). Overall, these changes resulted in a 1.67% ± 0.5 decline (mean change ± SE) in arborescent octocoral cover across FCR. In contrast, the octocoral density significantly increased in each region and in 4 of the 10 regional habitats from 2013 to 2023 overall, including three of the four habitats in Southeast Florida (SEFL) and the shallow forereefs in the Florida Keys (emmeans comparisons, *p* < 0.01; Figs. 1 and 3; Table S1). Octocoral density remained stable from 2013 to 2023 in all other regional habitats (*p* > 0.05). Collectively, the overall octocoral density increased by 33.7% ± 5.1 across FCR (mean change ± SE) from 2013 to 2023 (Fig. S1). The change in target species recruit density was most pronounced in the Florida Keys region, where it significantly increased in shallow and deep forereef habitats from 2013 to 2023 but decreased in patch reef habitats (Fig. 2; Table S1). Southeast Florida exhibited increases across all four habitats, while the Dry Tortugas showed increases in three habitats, however, increases across these seven regional habitats were non-significant (Table S1; *p* > 0.05). Overall, target species recruit density was higher at the end of the study period compared to the beginning across most of the Florida Coral Reef system.

## Spatiotemporal fluctuations in octocoral cover, adult density, and target species recruit density

Among the overall trends from 2013 to 2023, several interannual fluctuations in octocoral dynamics metrics varied by regional habitat and appeared to be linked to major disturbance events (Figs. 1 and 3; Table S1). Likely related to the 2014–2015 marine heatwave, octocoral cover significantly declined in the Florida Keys deep forereefs (*p* < 0.01) and in the Dry Tortugas patch and pinnacle reefs (*p* < 0.05). In SEFL, octocoral cover significantly declined in all four regional habitats following Hurricane Irma (2017–2018, *p* < 0.001). This decline was followed by significant year-to-year fluctuations on the nearshore ridge complex but gradual increases in cover until 2021/2022 on the inner, middle and outer reefs (Figs. 1 and 3, and S3). Similarly, in the Florida Keys, octocoral cover significantly decreased in all habitats following Hurricane Irma (*p* < 0.001) but gradually recovered, with a significant increase in cover on patch reefs from 2018 to 2019 (*p* < 0.001), shallow forereefs from 2019 to 2020 (*p* < 0.001), and deep forereefs from 2018 to 2019 (*p* < 0.01) and from 2020 to 2021 (*p* < 0.01). A significant decrease in cover was also observed in the Florida Keys patch reef from 2020 to 2021 (*p* < 0.01). A significant decrease in cover was also observed in the Florida Keys deep forereefs from 2022 to 2023, following Hurricane Ian (*p* < 0.001). In the Dry Tortugas, no significant declines in cover were observed following Hurricane Irma; however, a significant decrease in this region was observed on patch reefs between 2021 and 2022 (*p* < 0.001), which appeared unrelated to any major disturbance events. Moreover, significant declines in cover in all three Dry Tortugas habitats from 2022 to 2023 were observed (*p* < 0.001), which is likely also related to the combined impacts of Hurricane Ian in 2022 and the severe marine heatwave in 2023, which caused evident mortality during the 2023 surveys.

As with cover, octocoral density exhibited similar patterns following Hurricane Irma, with significant declines and subsequent recovery (Figs. 1 and 3). The density significantly decreased in the SEFL inner and outer reef habitats and Florida Keys shallow and deep forereefs from 2017 to 2018 (*p* < 0.001). In the aftermath, octocoral density recovered to pre-disturbance levels or exceeded them within two years, with significant increases from 2019 to 2020 on SEFL nearshore, middle and outer reefs (*p* < 0.001; Fig. 3), from 2018 to 2019 in both Florida Keys forereef habitats (*p* < 0.001), and from 2019 to 2020 in the Florida Keys shallow forereef (*p* < 0.001; Fig. 3). In contrast, no significant interannual fluctuations in octocoral density were observed in Dry Tortugas habitats during the study period.

Unexpectedly, year-to-year comparisons of target species recruit density revealed minimal changes following major disturbances (Figs. 2 and 3), which was partially due to high site-level variation. However, pairwise comparisons over 2- to 3-year intervals, and analysis of annual means against the overall mean value revealed significant differences over longer time periods, suggesting potential prolonged effects (Figs. S5 and S6, respectively).

Regional patterns varied considerably across FCR. In SEFL, target species recruit density significantly increased between 2017 and 2020 across all four habitats, followed by a marked decline in SEFL middle reefs between 2020 and 2023, while recruit density in the other three regions remained stable. In the Florida Keys patch reefs, recruit density significantly increased between 2013 and 2014, then significantly decreased from 2014 to 2018, followed by a marked increase between 2018 and 2020, and finally a significant decrease between 2020 and 2023 (Fig. S5). In the Florida Keys shallow and deep forereefs, the recruit density was significantly below average in most years between 2014 and 2017 (Fig. S6); however, significant increases occurred between 2016 and 2019 in both habitats. In the Florida Keys deep forereef, a significant decrease was also found between 2019 and 2023 (Fig. S5). Finally, in the Dry Tortugas pinnacle reefs, the recruit density significantly increased between 2016 and 2019, followed by significant decreases between 2019 and 2021 and 2019 and 2023 (Figs. 3 and S5). In the Dry Tortugas patch reefs, recruit density was significantly below average between 2022 and 2023 (Fig. S6).

The pronounced increases in recruit density across Florida’s coral reefs from 2017 to 2020 likely reflect post-Hurricane Irma recruitment dynamics in target species populations. However, subsequent declines between 2020 and 2023 may result from density-dependent effects or population self-thinning and/or the passage of Hurricane Ian (2022), specifically over the Dry Tortugas region.

## Discussion

Over the last several decades, benthic assemblages on Florida’s Coral Reef (FCR) have undergone significant shifts, primarily due to mass scleractinian mortality from repeated acute disturbances and limited recovery^11,16,26^. In contrast, we found considerable resilience in octocorals throughout FCR from 2013 to 2023, despite multiple acute disturbances. In some regional habitats, dramatic declines in both cover and density were recorded following Hurricane Irma in 2017; however, octocoral density recovered to or exceeded pre-disturbance values within one to two years (Fig. 1), likely driven by high rates of recruitment (Fig. 2). Most other regional habitat declines in cover appear to be related to marine heatwaves. While the data suggest that the 2014–2015 marine heatwave may have also suppressed recruitment, recovery in cover still occurred within two years in most locations. This recovery in cover likely reflects the capacity of surviving octocoral colonies to increase in size through enhanced branching and compensatory growth following partial mortality, potentially involving reallocation of energy from reproduction to growth^27,28^. These patterns align with studies from other Caribbean locations, which demonstrate that shallow-water octocorals can recover from acute disturbances and exhibit greater resilience to acute stressors than stony corals^11–14,16,17,29^.

In 2014 and 2015, the FCR experienced successive years of thermal stress as part of a global marine heatwave that resulted in widespread coral bleaching^30^. This caused significant declines among FCR scleractinian corals^16,31–33^. Despite reports of bleaching among octocorals in FCR^34^ and declines in octocoral cover in southeast Florida related to excess heat stress^35^, our results demonstrate that the octocoral density remained stable during the heatwave. Whereas octocoral cover did decline in some habitats such as the Dry Tortugas pinnacle habitats and on Florida Keys deep forereefs from 2014 to 2015, recovery was fast. This depth-specific response may reflect reduced thermal tolerance in deeper octocoral communities that are less acclimatized to temperature extremes^36^, as well as potential differences in species community composition and timing of mortality responses between shallow and deep habitats^37^. These results support the understanding of octocorals as resilient following thermal stress^34^, particularly in comparison with scleractinians, but not resistant^38^. Octocoral colonies, like scleractinian colonies, can suffer whole and partial mortality [e.g.,^39,40^]. Partial mortality is not captured by colony density but may be captured by cover estimates. Effects from the 2014–2015 marine heatwave were also observed in target species recruit density, which declined in most (8 out of 10) regional habitats between 2014 and 2016 (Fig. 2), suggesting either a pause in recruitment or mortality of recruits during this period. The effects of marine heatwaves on octocoral reproductive performance and recruitment success are not well documented and are likely to be species specific [e.g.,^41^]. Moreover, how other indirect effects that may influence octocoral recruitment during marine heatwaves, such as increases in the abundance of macroalgae or cyanobacteria, have not been well studied. Consequently, demographic assessments are needed to understand the extent to which disturbances influence octocoral colony size, health, reproductive output, and recruitment dynamics.

The other major marine heatwave during our study period occurred in the final year, 2023, closely following Hurricane Ian. This heatwave was the most extreme heatwave that FCR experienced and was significantly hotter than the 2014–2015 marine heatwave, with scleractinian bleaching occurring as early as June^42,43^. As with the 2014 and 2015 marine heatwaves, we observed declines in octocoral cover on Florida Keys deep forereefs and within all three Dry Tortugas habitats from 2022 to 2023, which were surveyed in August 2023, the last of all the regions. Heat-related mortality was observed during Dry Tortugas surveys, supporting reports of thermal thresholds for octocoral survival^44^. However, fully attributing this decline to the heatwave is difficult because category 4 Hurricane Ian tracked just west of the Dry Tortugas between the 2022 and 2023 surveys. The declines in octocoral density in the Florida Keys shallow forereef habitats and in cover in the deep forereefs from 2022 to 2023 may be related to either (or both) Hurricane Ian or the 2023 marine heatwave because heat-related mortality was also observed during the July Florida Keys surveys. Surveys conducted in a subset of the Florida Keys sites in early 2024 indicated that the 2023 marine heatwave resulted in drastic declines in octocoral density, particularly in patch reef habitats, where in situ temperatures were higher than those of forereefs (Stein et al. 2024, see also unpub. data at https://geodata.myfwc.com). Continued long-term monitoring of octocorals is needed to understand whether octocorals can recover, as they have from previous disturbances, including the 2010 cold-water event^23^, or whether mass mortality has reduced populations beyond critical thresholds^45^.

Spatiotemporal variations in target species recruit density during our study period demonstrate the role of recruitment in the recovery of octocoral densities across FCR^38^. Hurricane Irma, which made landfall in the Lower Florida Keys in September 2017, resulted in a significant decline in arborescent octocoral cover in all seven habitats in the Florida Keys and SEFL; density significantly declined between 2017 and 2018 in four out of seven of these habitats as well. This was followed by a marked increase in recruit density, resulting in higher numbers of small colonies (< 5 cm) across all regional habitats between 2019 and 2020 compared with those reported in 2017 and 2018 (Fig. 2 and supplementary material). A surge in recruitment of western Atlantic and Caribbean octocorals following acute disturbance has been previously documented. For example, Yoshioka^47^ attributed the dramatic increase in recruit density on the southwest coast of Puerto Rico in 1984 and 1985 to the massive die-off of *Diadema antillarum* that occurred in 1984^46,47^. Similar findings have been reported in St. John, USVI, where octocoral recruits played a central role in octocoral recovery following two major hurricanes (Irma and Maria). Recruit density initially decreased two months after the hurricanes before returning to pre-disturbance levels by 2019^29^. While not all of our annual surveys detected declines in the density of recruits immediately following Hurricane Irma, it is important to mention that our surveys only collected data on several common target species, whereas the surveys at St. John encompassed all species in the community. In addition, correlation analysis of density trends between total octocoral and target species populations reveals strong to moderate relationships across all FCR habitats during the study period (Table S2, Fig. S7), supporting the use of target species as proxies for broad-scale patterns across FCR. FCR is known to be a well-connected system with high potential for larval exchange^48^, suggesting that the increased recruitment observed between 2018 and 2020, particularly in severely impacted locations, may have been supported by larvae from other FCR habitats or potentially imported from elsewhere in the Caribbean^49^. Overall, recruitment appears to have facilitated recovery on FCR following Hurricane Irma, underscoring its importance to western Atlantic octocorals.

Our analysis of octocoral cover revealed fluctuating interannual changes on FCR, with significant increases and decreases occurring between years. Although octocoral cover declined significantly between 2013 and 2023 in 6 out of the 10 regional habitats (Table S1), at least partially due to the study concluding following multiple disturbances, it remained similar to previously reported ranges for this region and still vastly exceeded that of scleractinians. For example, cover on Florida Keys shallow forereefs in 2023 exceeded the maximum cover recorded between 2000 and 2009, whereas on deep forereefs and patch reefs, it remained consistent with the previously reported range^11^. Given that octocoral populations in the Florida Keys were significantly impacted by the 2010 cold-water event^23^, their ability to recover to pre-disturbance cover percentages as early as 2013 and again thereafter multiple disturbances through 2023 is additional evidence for their recovery potential. Similarly, the octocoral cover on the SEFL and Dry Tortugas reefs in this study was consistent with previous records from 2004, except for the SEFL outer reef habitats, where cover has declined substantially since 2004^16^. It is plausible that some variability may be introduced by noise from sampling techniques, but changes in cover, but not density, may reflect processes such as partial colony mortality, colony shrinkage, or the loss of adults, which have important ecological consequences for resilience and require further study. We recommend that, in addition to cover, long-term monitoring studies on Caribbean octocorals incorporate species- or community-resolution demographic metrics, such as biomass proxies^20,50,51^ or size-frequency distributions^38^, to further clarify the ecological processes at play on these reefs.

Certain caveats limit the ability of this study to provide a more thorough understanding of octocoral dynamics during our study period. First, we recognize that changes in community composition likely occurred during our study period, particularly following disturbance events. For example, while the observed trends in total octocoral cover and density following the 2014–2015 marine heatwave or Hurricane Irma (2017) could have been consistent across populations, certain species likely fared better than others did, as was documented in the USVI^13,15^. Second, the observed declines in octocoral cover may be related to the loss of colonies or species that create large canopies and may not have experienced the recovery from recruitment observed in our target species population, which was limited to five target species in the Florida Keys and Dry Tortugas and three species in SEFL. With such a small set of target species relative to the overall richness of octocorals in the FCR (> 50 species^52^, , we were not able to fully capture the processes shaping the dynamics of all octocoral populations. These differences can be attributed to species-specific bleaching susceptibilities^40^; variable growth rates among species or branch thickness^53^; and/or distinct life-history traits^54,55^. Finally, our dataset did not account for the presence of encrusting octocoral species. While only two encrusting species are found across the FCR (*Erythropodium caribaeorum* and *Briareum asbestinum*), their cover and density can be relatively high in some locations and may function in the ecosystem differently than canopy-forming arborescent species do. Together, considerably more work is needed to determine octocoral species-specific variability across regional habitats, which would increase the accuracy of evaluating their current and future trends.

Overall, this study highlights that arborescent octocorals in FCR are capable of recovering from acute disturbances, particularly physical disturbances such as hurricanes, a process that is likely driven by strong temporal increases in recruitment in populations of several common octocoral species. These trends contrast with those of stony coral communities on the FCR, which are increasingly impacted by bleaching events^31,43^, disease^56–58^, and the low recruitment success of major reef-building taxa^59^. While octocoral dominated reefs may represent the “new normal” for shallow Caribbean reefs^15,34^, how populations of octocorals on FCR recover from the 2023 marine heatwave has yet to be determined. This study provides evidence that recruitment can support the rapid recovery of octocoral density following disturbance; however, recovery of cover is a slower process, requiring time for recruits to mature into large, canopy-forming colonies. While octocorals demonstrated remarkable resilience during our study period, the increasing frequency and severity of disturbance events may ultimately exceed even octocorals’ capacity to recover.

## Materials and methods

### Study location

**Fig. 4:**
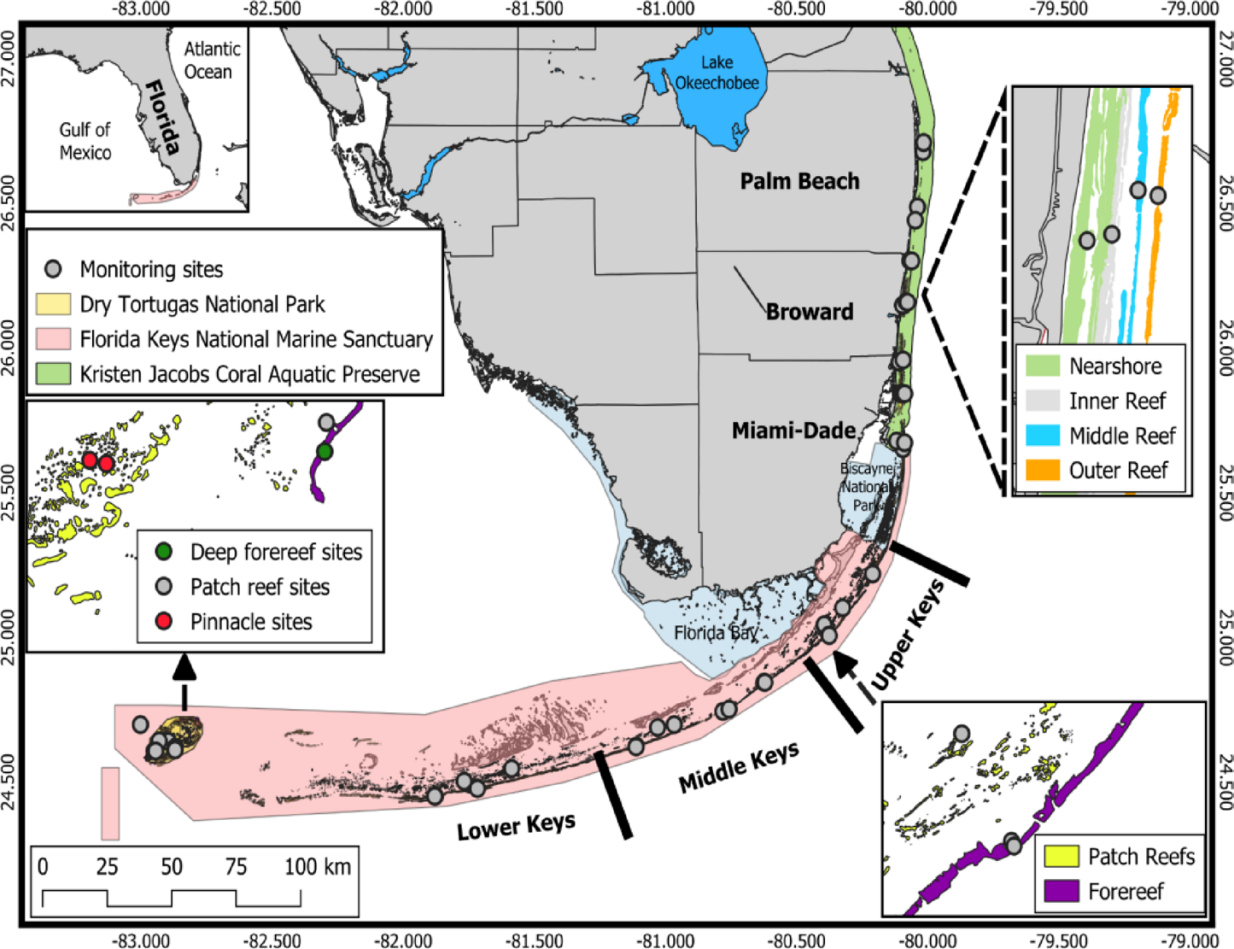
Main - Florida’s Coral Reef (FCR), study sites, coral reef habitats and management authorities of each region. Inset top left – an overview of the Florida Peninsula. Other insets indicate regional reef habitats in Southeast Florida (SEFL; top right), the Florida Keys (FK; bottom right), and the Dry Tortugas (DRTO; left). This figure was created using QGIS v3.40 www.qgis.org^65^.

Florida’s Coral Reef extends 595 km from Martin County in southeast Florida to the Dry Tortugas (Fig. 4). It comprises three regions: high-latitude coral communities offshore from southeastern Florida (SEFL), the limestone island chain of southern Florida known as the Florida Keys, and the comparatively isolated Dry Tortugas, another group of islands located 113 km west of the Florida Keys^60–63^. SEFL reefs lie near the northern limit of coral distribution in the tropical western Atlantic, extending from the St. Lucie inlet in Martin County to Biscayne Bay, and are contained within the Kristen Jacobs Coral Aquatic Preserve. The Florida Keys lie within the Florida Keys National Marine Sanctuary, which protects over 9,800 km^2^ of water surrounding the Keys and the Dry Tortugas, excluding the Dry Tortugas National Park. The Dry Tortugas National Park, located 113 km west of Key West, protects over 260 km^2^ of water and is the only of the three protected areas that includes an exclusion zone prohibiting fishing and anchoring.

Our study included 45 sites stratified across sub-regions and habitats (Fig. 4). In SEFL (*n* = 18 total sites), the sub-regions include Palm Beach County (*n* = 4), Broward County (*n* = 6), and Dade County (*n* = 8). In the Florida Keys (*n* = 20 total sites), the sub-regions are the Lower (*n* = 6), Middle (*n* = 8), and Upper Keys (*n* = 6). The Dry Tortugas region (*n* = 7 sites) is not subdivided into additional sub-regions. Our analysis also included multiple reef habitats. SEFL is composed of three shore-parallel linear reef ridges and a nearshore ridge complex that increase in depth as they increase in distance from the shoreline^64^: nearshore (*n* = 2) and inner (*n* = 5) reefs (6–8 m depth each), middle (*n* = 4, 12–14 m), and outer reefs (*n* = 7, 18 m). All four ridges lie within 3 km of a highly urbanized coastline with heavy maritime use. Within the Keys, patch reefs (*n* = 6, 2–10 m), shallow forereefs (*n* = 7, 2–7 m), and deep forereefs (*n* = 7, 11–16 m) are found across all three sub-regions. Within the Dry Tortugas, patch reefs (*n* = 2, 5–10 m), pinnacle reefs (seamounts with steep walls; *n* = 3, 6–13 m), and deep forereefs (*n* = 2, 14–22 m) were included in the analysis. Despite similar nomenclature, patch reefs and deep forereefs in the Keys and Dry Tortugas are not considered equivalent habitats because of differences in the geological structure of these regions. Thus, all habitats are considered regional habitats.

## Data collection

We used data collected annually at permanent monitoring sites within the Coral Reef Evaluation and Monitoring Project (CREMP) in the Florida Keys, the Southeast Florida Coral Reef Evaluation and Monitoring Project (SECREMP) in SEFL, and the Dry Tortugas Coral Reef Evaluation and Monitoring Project (DTCREMP). These projects were established to provide local resource managers with annual updates on the status of the coral reefs in the region. From these projects, we used metrics of arborescent octocoral cover, density, and target species recruit density (derived from colony height data) beginning in 2013, the first year when all three metrics were recorded at all the examined sites, through 2023, for a total of 11 years.

Annual surveys were conducted at all 45 sites in the summer months (May–September and sometimes later, depending on weather). Each site consists of four ~ 22 m belt transects (except for one patch reef site in the Dry Tortugas with three transects) marked with permanent stakes installed in the reef at each end. For octocoral cover, consecutive images, each ~ 40 cm wide, were taken at a fixed distance from the substratum along the entire ~ 22 m belt transect and analyzed using PointCount ’99^66^. Random points were overlaid on each image, and the taxonomic group directly beneath each point was identified; octocoral cover was recorded as either arborescent or encrusting, but only arborescent octocoral cover was analyzed here to align with counts of arborescent octocoral density. The methods used to collect images and estimate percent cover are described in previous studies more detail in earlier publications [e.g., ^11^,^67^]. For octocoral density, all arborescent octocorals were counted within the first 10 × 1 m of the four transects, based on whether any portion of their holdfast was present within the transect area. Due to the fragmented growth form of encrusting species and their potential for tissue reconnection, only arborescent species were included in octocoral density surveys. This includes the erect form of *Briareum asbestinum*.

To assess the role of recruitment in the observed density trends, we analyzed the number of octocoral recruits per m^2^ within the population of five target species in the Florida Keys and Dry Tortugas: *Pseudoplexaura porosa*, *Antillogorgia bipinnata*,* Antillogorgia americana*,* Gorgonia ventalina*, and *Eunicea flexuosa*; only the last three species were examined on SEFL reefs. These species were selected because they can be reliably distinguished in the field and are relatively common in these reef habitats. The maximum heights of all colonies of each target species were measured within the first 10 × 1 m of the four transects by holding the 0 cm end of a 0.5 m PVC measuring stick at the lowest point of the holdfast on the substratum and sweeping the axis and branches up and alongside the stick. Colonies that were ≤ 5 cm in height were classified as recruits following research by^68^. Recruit density was calculated by dividing the total number of target species recruits by the transect area (10 m^2^) for each transect at all 45 sites annually.

### Statistical analysis

To assess long-term patterns in the octocoral distribution, GLMMs were created for octocoral cover, density, and recruit density using the “glmmTMB” package in R^69,70^. For each response variable, multiple candidate models were created to assess how each variable changed temporally and the predominant spatial scale at which it varied over the FCR. Multiple candidate models were created, each containing the sample year and at least one of the spatial predictors: region, sub-region, depth, and regional habitat, which were fitted as fixed categorical factors (Table 1). The sample year was incorporated as a fixed categorical predictor so that the results were not skewed by the first or last sample year and to allow for between-year comparisons to be explored further during post hoc analysis. The minimum adequate model was selected from the candidate models using the Akaike information criterion (AIC;^71^. Octocoral density was fitted as a negative binomial GLMM (log link) after overdispersion was detected in the data; the cover was fitted as a binomial GLMM (logit link), with the number of random points on each transect accounted for using weights; recruit density was fitted as a negative binomial GLMM (log link). The hierarchical structure of the data was accounted for in all the models by fitting a random intercept of transects (stations) nested within each site.

The models were validated using the “DHARMa” package in R^72^, with residual diagnostics conducted on the fitted models for overdispersion, heterogeneity, and temporal autocorrelation. Temporal autocorrelation was identified for the density GLMM, where data points in consecutive years were more similar than expected and violated independence, and a first-order autoregressive correlation structure was fitted. Model validation revealed no further problems. Post hoc pairwise assessment of the retained factors within the minimum adequate models was conducted using the “emmeans” package in R^73^. Differences were considered significant when *p* < 0.05. Temporal changes were assessed independently at the regional scale (i.e., 2013 vs. 2014 in the Florida Keys) during post hoc analysis.

To validate that target species density represents total arborescent octocoral community dynamics, we conducted supplementary analyses comparing temporal trends between total and target species densities across regional habitats. Linear regression models examined annual density changes (slopes) for both groups from 2013 to 2023, with slope differences assessed using ANCOVA-type approaches. Pearson and Spearman correlation coefficients were calculated for each regional habitat to quantify relationship strength between total and target species densities. Statistical significance was evaluated at α = 0.05.

## Supplementary Information

Below is the link to the electronic supplementary material.

Supplementary Material 1

Supplementary Material 2

## Data Availability

Data from the Coral Reef Evaluation and Monitoring Project (CREMP) in the Florida Keys, the Southeast Florida Coral Reef Evaluation and Monitoring Project (SECREMP), and the Dry Tortugas Coral Reef Evaluation and Monitoring Project (DT CREMP) are available for download here: https://myfwc.com/research/habitat/coral/cremp/data. All other data supporting the findings of this study are provided in Supplementary Information. R codes used for statistical analyses and data visualization are available on https://github.com/ronenliberman/Florida_Octocoral_forests.
