# Supplementary material for: Octocoral dynamics over a decade on Florida’s coral reef
Source: Sci Rep. 2025 Oct 28;15:37635. doi: 10.1038/s41598-025-21610-5 (PMC12569018; doi:10.1038/s41598-025-21610-5)
Supplement: Supplementary file 2 — Supplementary Material 2 [file 41598_2025_21610_MOESM2_ESM.docx]

**Octocoral dynamics over a decade on Florida’s Coral Reef**

Ronen Liberman^1^**^*^**, Alexandra Hiley^1^, Lindsay K. Huebner^2^, Michael A. Colella^2^, Rob R. Ruzicka^2^, David S. Gilliam^1^, Nicholas P. Jones^1^

^1^ National Coral Reef Institute, Nova Southeastern University.

^2^ Fish & Wildlife Research Institute, Florida Fish & Wildlife Conservation Commission

**Supplementary material**

Table S1. Comparison of octocoral metrics between 2013 and 2023 across regional habitats. Mean values (± SE) for octocoral cover, density, and recruit density at the beginning (2013) and end (2023) of the study period. Bold values indicate statistically significant differences between years based on emmeans post-hoc comparisons of fitted GLMMs (p < 0.05).

| **Region** | **Habitat** | **Octocoral cover (%)** | | **Octocoral density (m^2^)** | | **Recruit density (m^2^) *** | |
| --- | --- | --- | --- | --- | --- | --- | --- |
|  |  | 2013 | 2023 | 2013 | 2023 | 2013 | 2023 |
| **Southeast Florida** | Nearshore | **0.8±0.01** | **0.07±0.01** | **10.85±1.2** | **16.89+1.59** | 0.17±0.09 | 0.31±0.26 |
|  | Inner | 0.07±0.02 | 0.06±0.01 | 5.74±0.73 | 8.29±0.9 | **0.09±0.09** | **0.26± 021** |
|  | Middle | 0.05±0.01 | 0.06±0.005 | **5.85±0.7** | **10.2±1.16** | 0.19±0.22 | 0.24±0.17 |
|  | Outer | **0.13±0.01** | **0.11±0.01** | **14.5±1.16** | **18.01±1.36** | **0.06±0.08** | **0.16±0.12** |
| **Florida Keys** | Patch | 0.18±0.01 | 0.19±0.02 | 12.03±1.22 | 12.81±1.22 | 0.49±0.46 | 0.34±0.32 |
|  | Shallow | **0.17±0.01** | **0.14±0.01** | **15.15±2.36** | **23.09±3.48** | 0.53±0.4 | 0.7±0.43 |
|  | Deep | **0.09±0.006** | **0.07±0.006** | 14.49±1.02 | 16.7±1.76 | 0.62±0.3 | 0.79±0.43 |
| **Dry Tortugas** | Patch | 0.11±0.03 | 0.06±0.01 | 6.8±1.89 | 7.81±1.99 | 0.07±0.15 | 0.33±0.18 |
|  | Pinnacle | **0.13±0.02** | **0.10±0.01** | 10.65±1.98 | 9.67±2 | 0.35±0.11 | 0.2 ±0.12 |
|  | Deep | **0.13+0.02** | **0.07±0.006** | 13.5±2.49 | 20.12±4.06 | 3.5±1.06 | 0.3±0.17 |

Table S2. Correlation between target species and total octocoral density by habitat. Pearson and Spearman correlation coefficients (2013-2023) showing strong to moderate positive associations across all habitats (all p < 0.001). N represents the number of observations per habitat.

| **Habitat** | **Pearson r** | **Spearman r** | **P value** | **Correlation strength** | **N observations** |
| --- | --- | --- | --- | --- | --- |
| **SEFL Inner** | 0.568 | 0.617 | < 0.001 | Moderate | 132 |
| **SEFL Nearshore** | 0.716 | 0.701 | < 0.001 | Strong | 88 |
| **SEFL Middle** | 0.696 | 0.705 | < 0.001 | Moderate | 132 |
| **SEFL Outer** | 0.465 | 0.432 | < 0.001 | Moderate | 264 |
| **FK Patch** | 0.978 | 0.969 | < 0.001 | Strong | 264 |
| **FK Shallow Forereef** | 0.747 | 0.749 | < 0.001 | Strong | 300 |
| **FK Deep Forereef** | 0.864 | 0.82 | < 0.001 | Strong | 301 |
| **DRTO Patch** | 0.496 | 0.634 | < 0.001 | Moderate | 77 |
| **DRTO Pinnacle** | 0.934 | 0.934 | < 0.001 | Strong | 132 |
| **DRTO Deep Forereef** | 0.985 | 0.98 | < 0.001 | Strong | 44 |


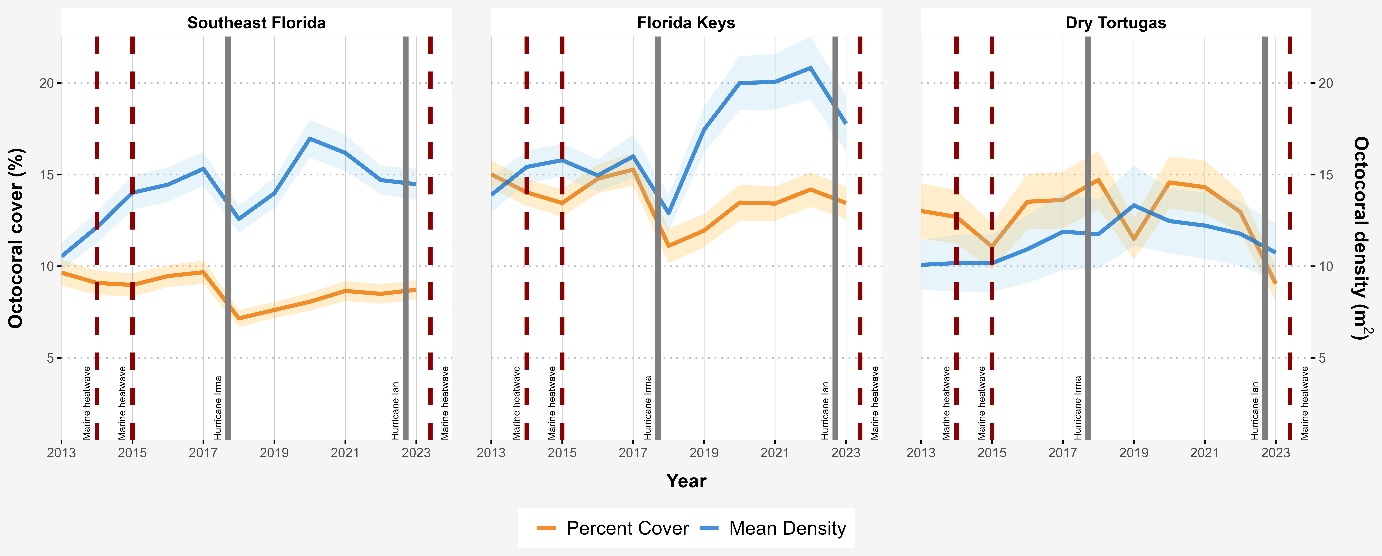


**Figure S1.** Arborescent octocoral mean (± SE) percent cover (orange) and density (blue) from 2013 to 2023 in 3 regions on Florida’s Coral Reef: Southeast Florida, Florida Keys, and Dry Tortugas. Major disturbance events, including marine heatwaves and hurricanes, are indicated using vertical dashed and solid lines, respectively.


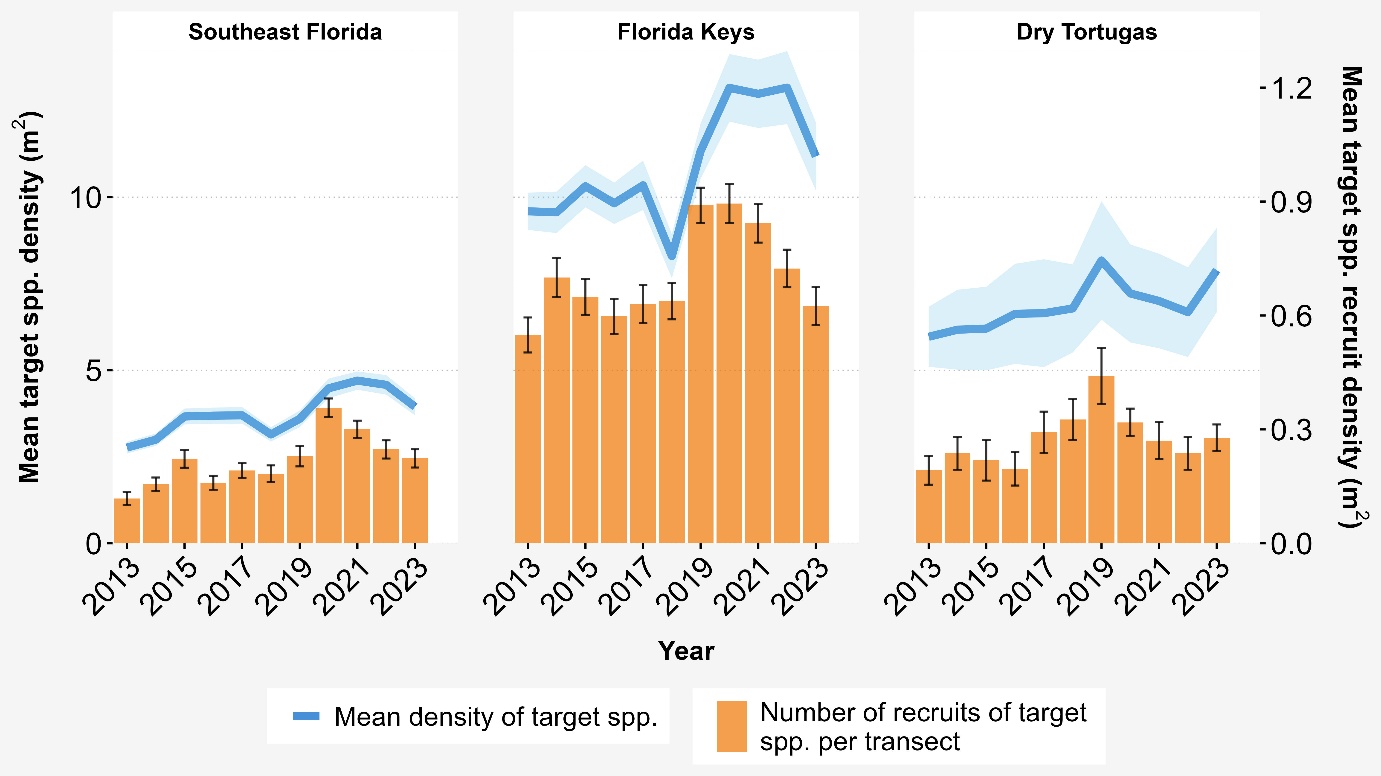


**Figure S2.** Mean (± SE) density of target octocoral species (blue) and mean target species recruit density (≤ 5 cm height; orange) from 2013 to 2023 in 3 regions on Florida’s Coral Reef: Southeast Florida, Florida Keys, and Dry Tortugas. In the Florida Keys and Dry Tortugas, target species included *Pseudoplexaura porosa, Antillogorgia bipinnata, Antillogorgia americana, Gorgonia ventalina,* and *Eunicea flexuosa*; only the last three species were targeted in SEFL. Major disturbance events, including marine heatwaves and hurricanes are indicated using vertical dashed and solid lines, respectively.

**
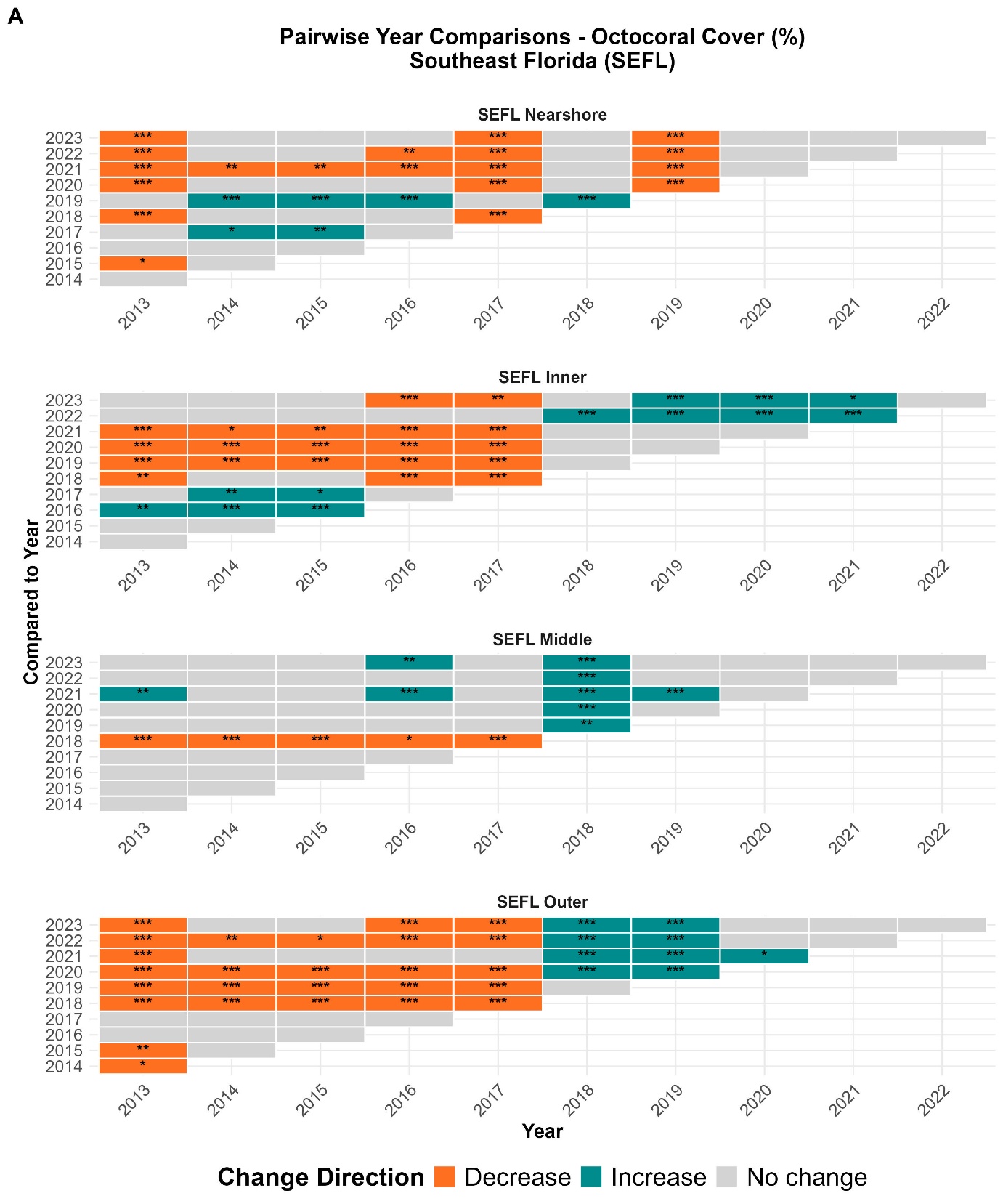

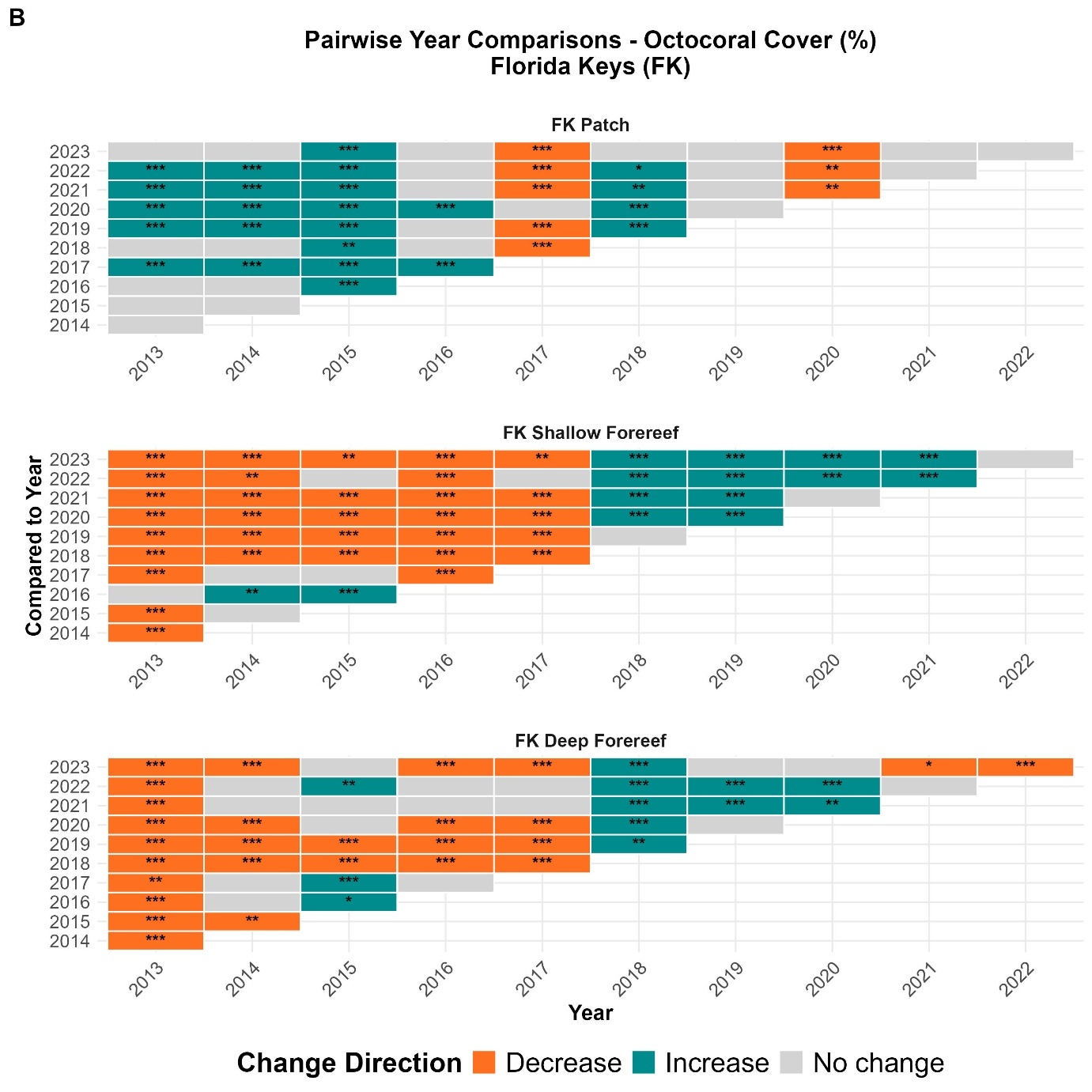

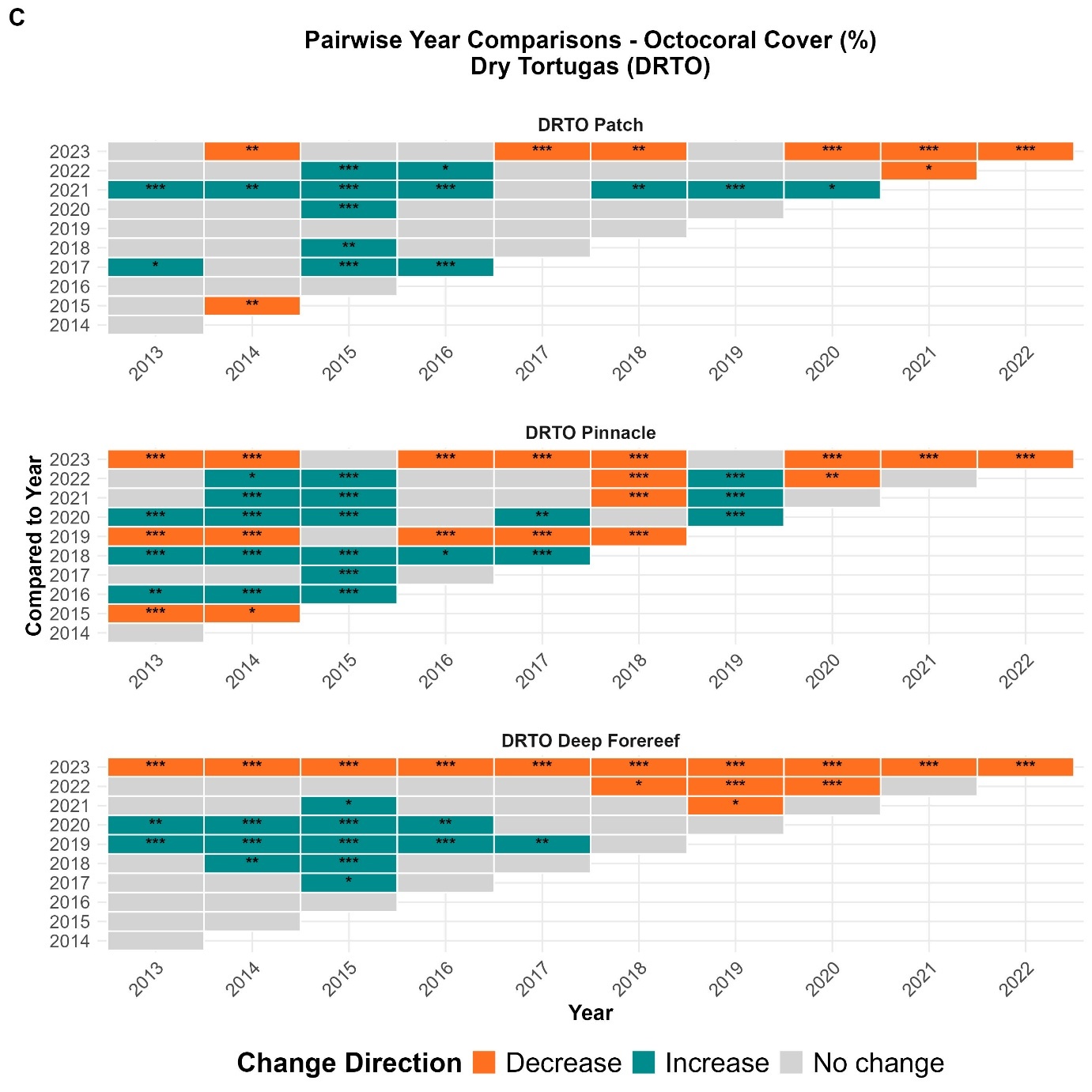
** **Figure S3.** Pairwise year-to-year comparisons of cover of arborescent octocorals from 2013 to 2023 in 10 regional habitats on Florida's Coral Reef, displayed as a matrix heatmap. (A) Southeast Florida (SEFL), (B) Florida Keys (FK), and (C) Dry Tortugas (DRTO). Each cell represents a pairwise comparison between years (x-axis vs y-axis), with colors indicating the direction of significant change: teal for increase, orange for decrease, and gray for no significant change. Asterisks denote significance levels (* p < 0.05, ** p < 0.01, *** p < 0.001). Depth increases top to bottom in each panel, but comparable depths are found between SEFL nearshore and inner reefs and between FK patch and shallow forereefs. Comparisons are based on the best-fit model and computed by the emmeans package in R.

**
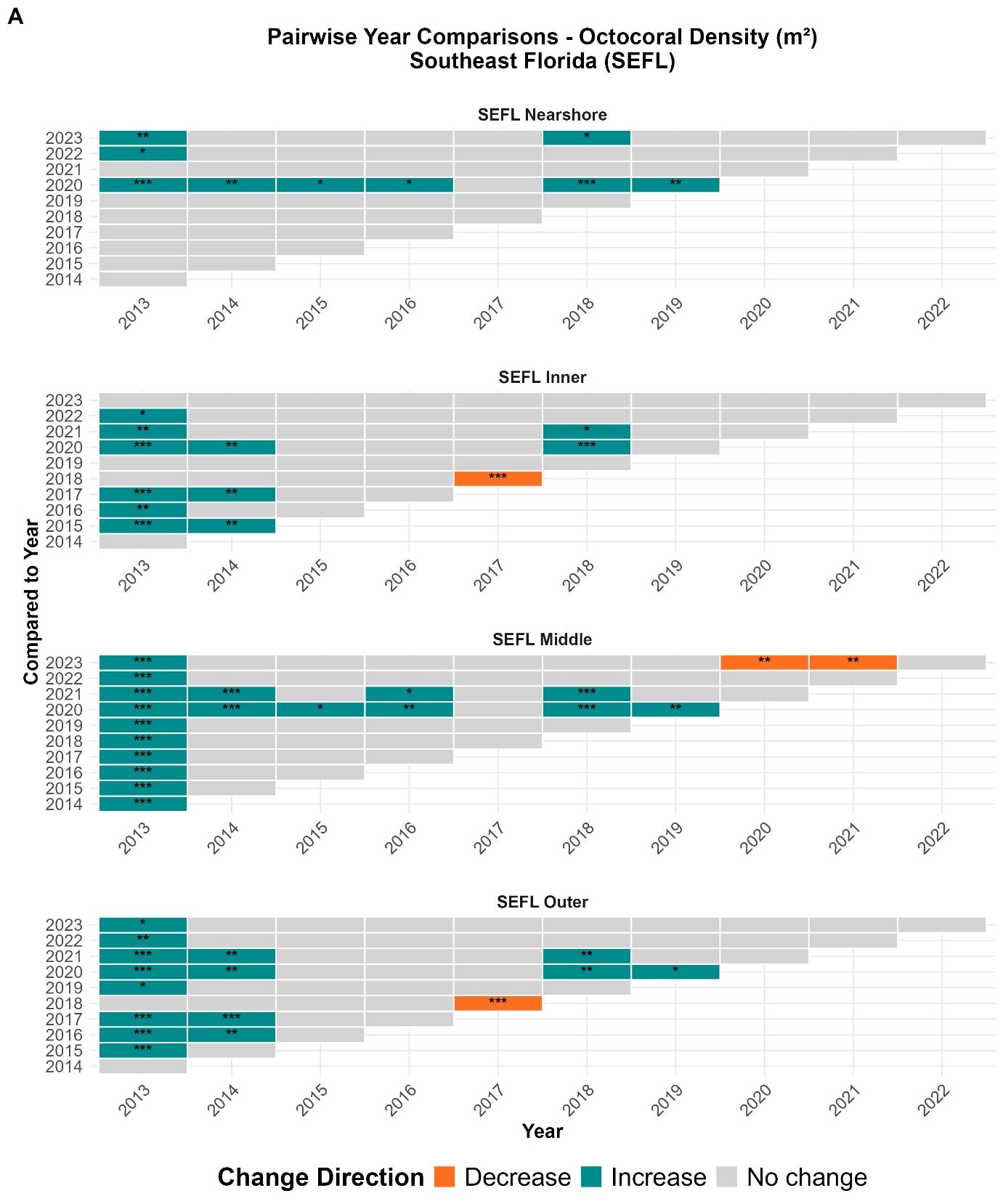

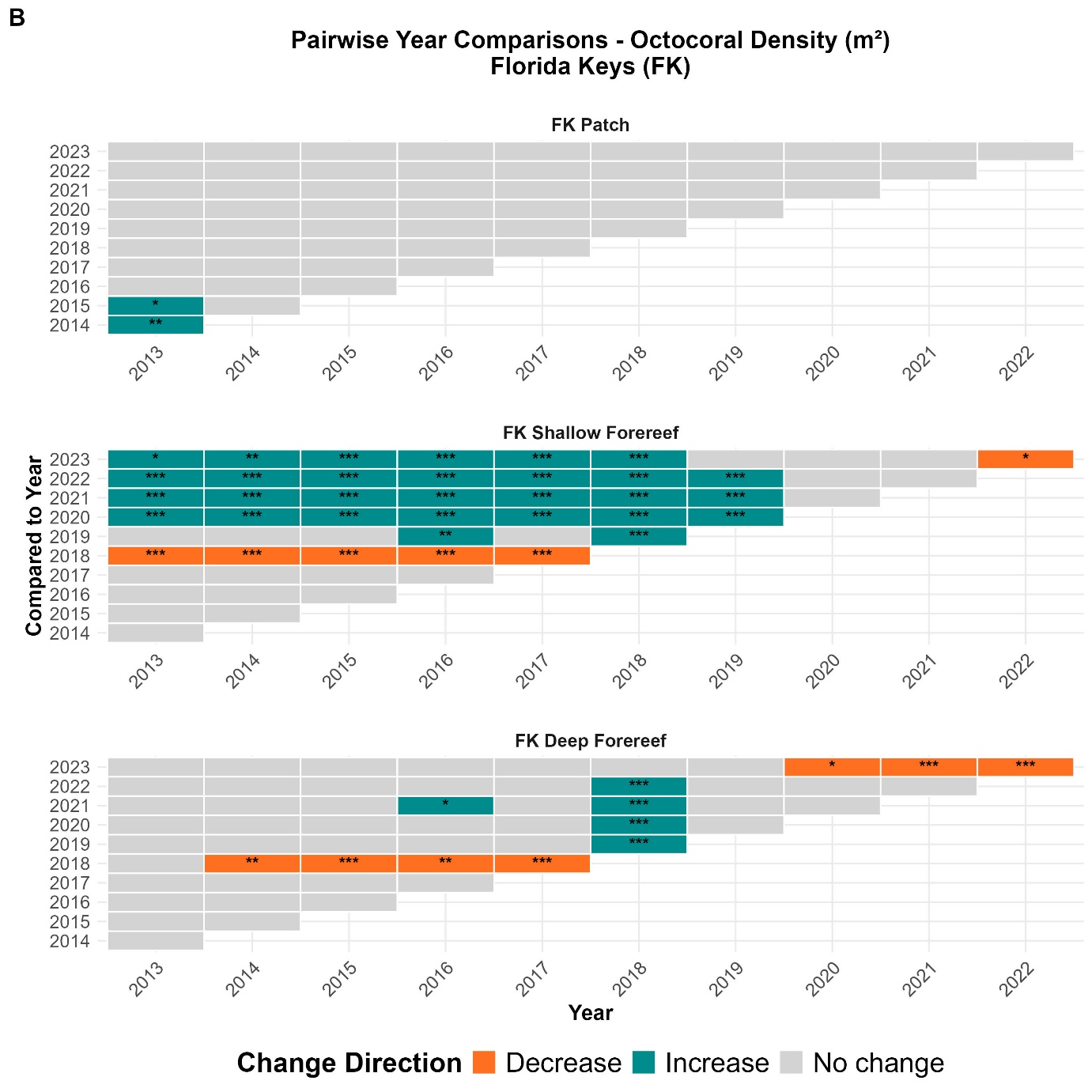

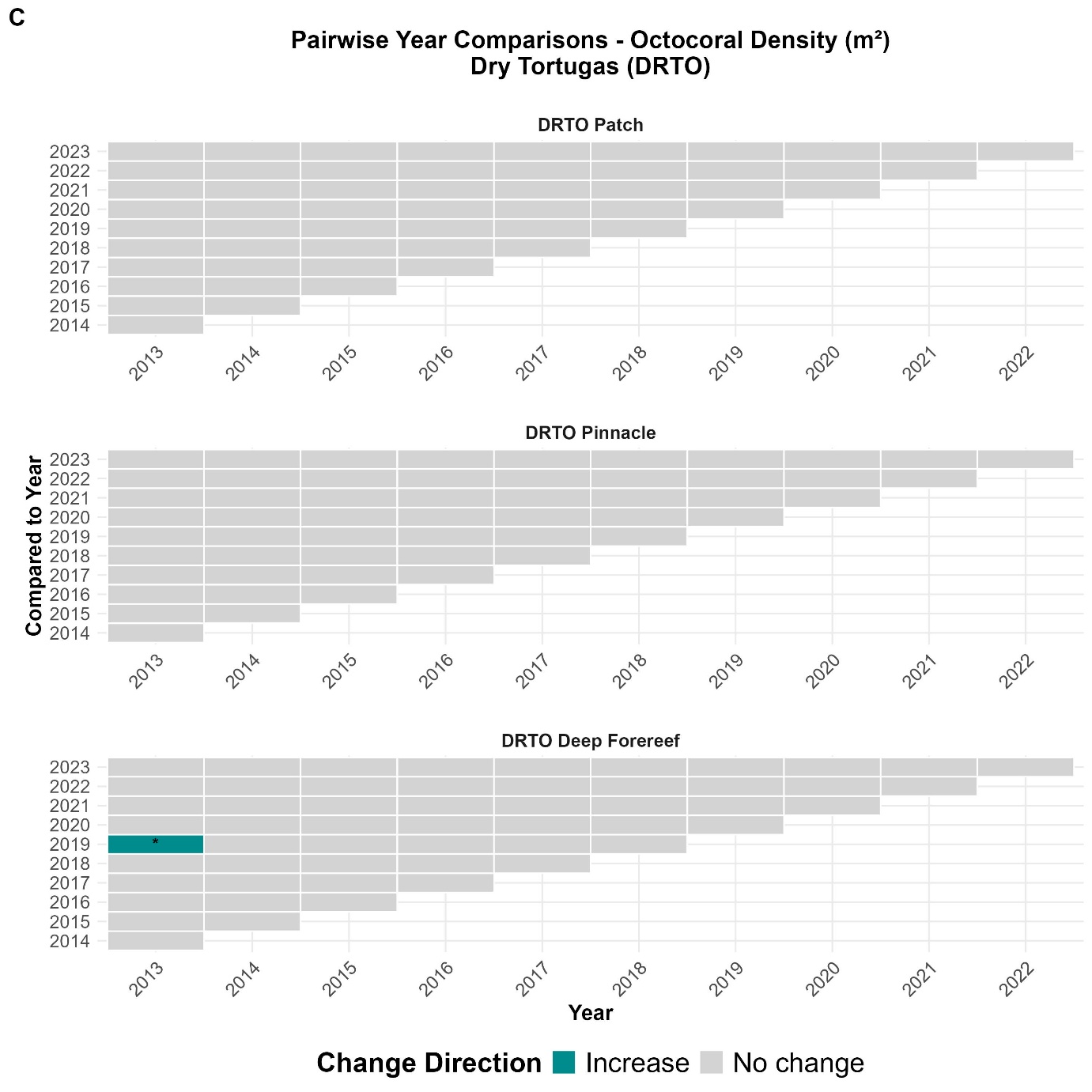
**

**Figure S4.** Pairwise year-to-year comparisons of density of arborescent octocorals from 2013 to 2023 in 10 regional habitats on Florida's Coral Reef, displayed as a matrix heatmap. (A) Southeast Florida (SEFL), (B) Florida Keys (FK), and (C) Dry Tortugas (DRTO). Each cell represents a pairwise comparison between years (x-axis vs y-axis), with colors indicating the direction of significant change: teal for increase, orange for decrease, and gray for no significant change. Asterisks denote significance levels (* p < 0.05, ** p < 0.01, *** p < 0.001). Depth increases top to bottom in each panel, but comparable depths are found between SEFL nearshore and inner reefs and between FK patch and shallow forereefs. Comparisons are based on the best-fit model and computed by the emmeans package in R.


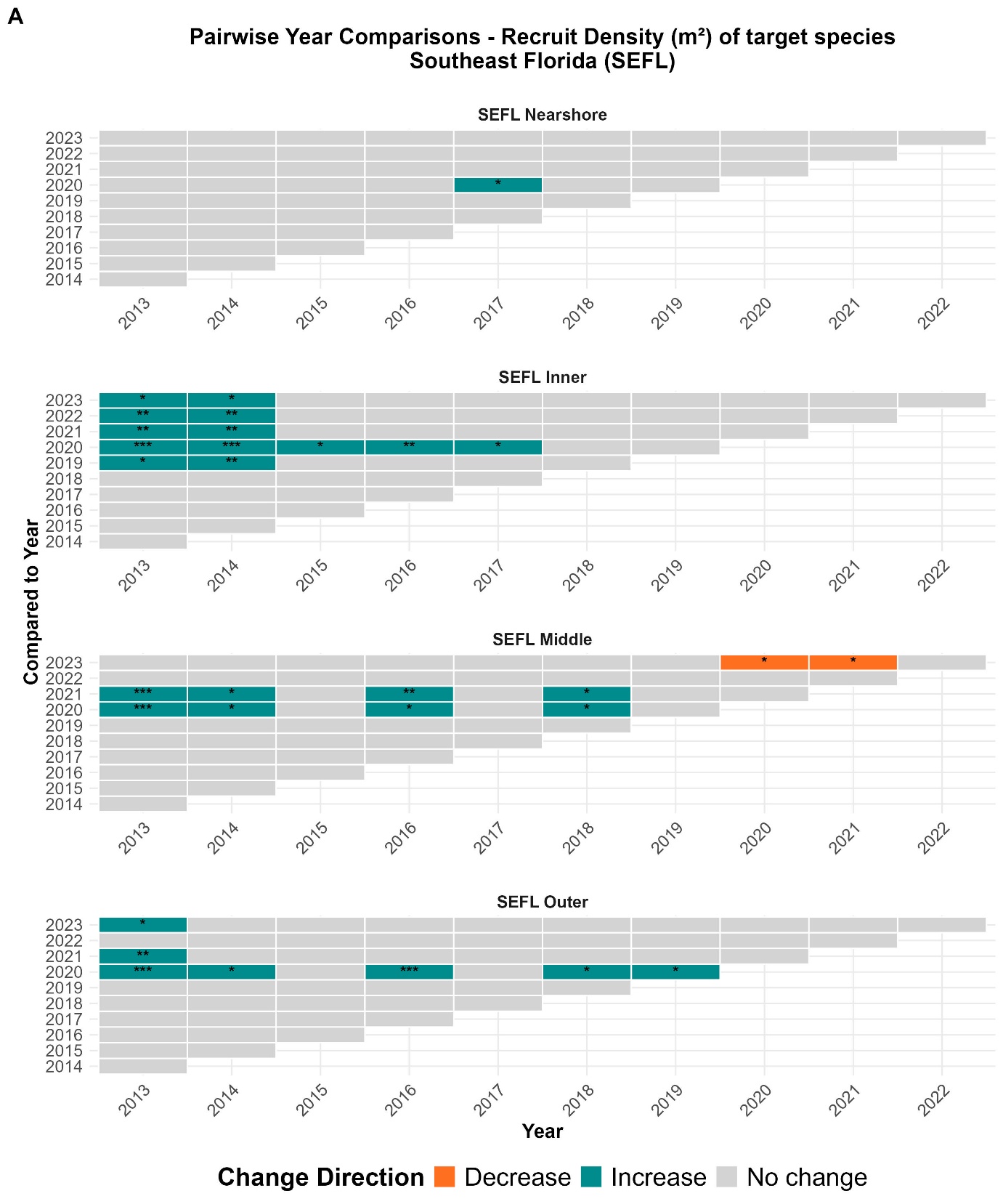


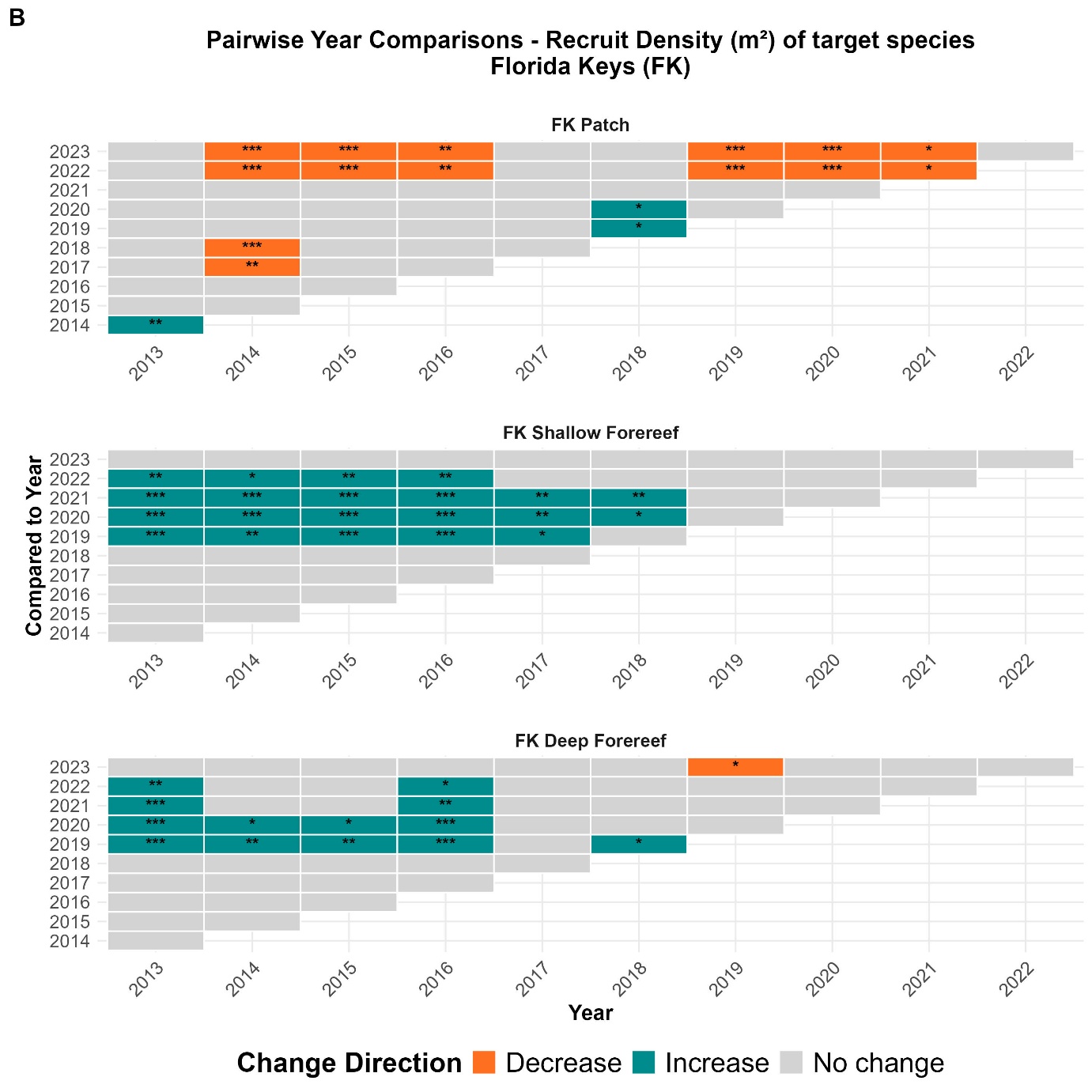

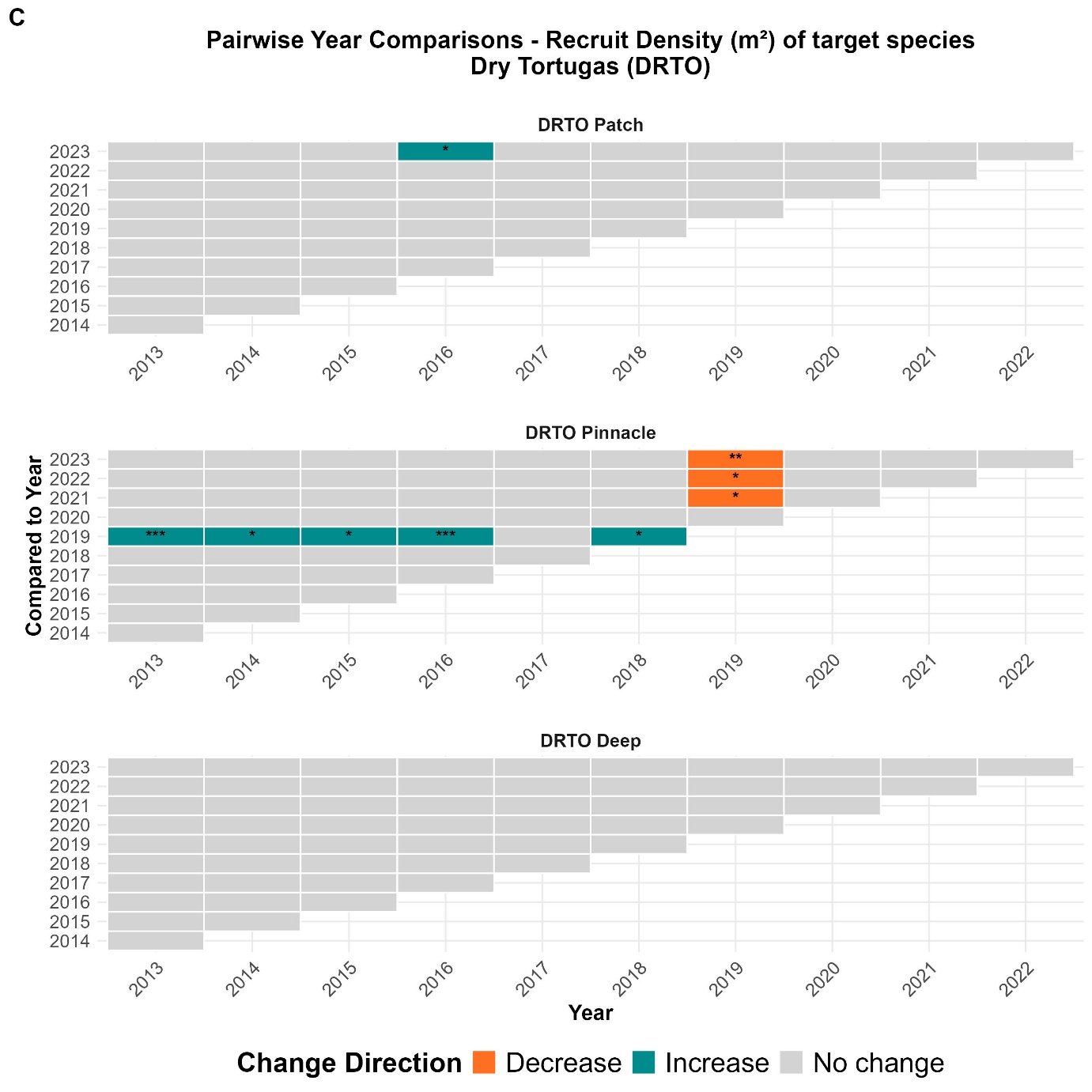


**Figure S5.** Pairwise year-to-year comparisons of target species recruit density of from 2013 to 2023 in 10 regional habitats on Florida's Coral Reef, displayed as a matrix heatmap. (A) Southeast Florida (SEFL), (B) Florida Keys (FK), and (C) Dry Tortugas (DRTO). Each cell represents a pairwise comparison between years (x-axis vs y-axis), with colors indicating the direction of significant change: teal for increase, orange for decrease, and gray for no significant change. Asterisks denote significance levels (* p < 0.05, ** p < 0.01, *** p < 0.001). Depth increases top to bottom in each panel, but comparable depths are found between SEFL nearshore and inner reefs and between FK patch and shallow forereefs. Comparisons are based on the best-fit model and computed by the emmeans package in R.


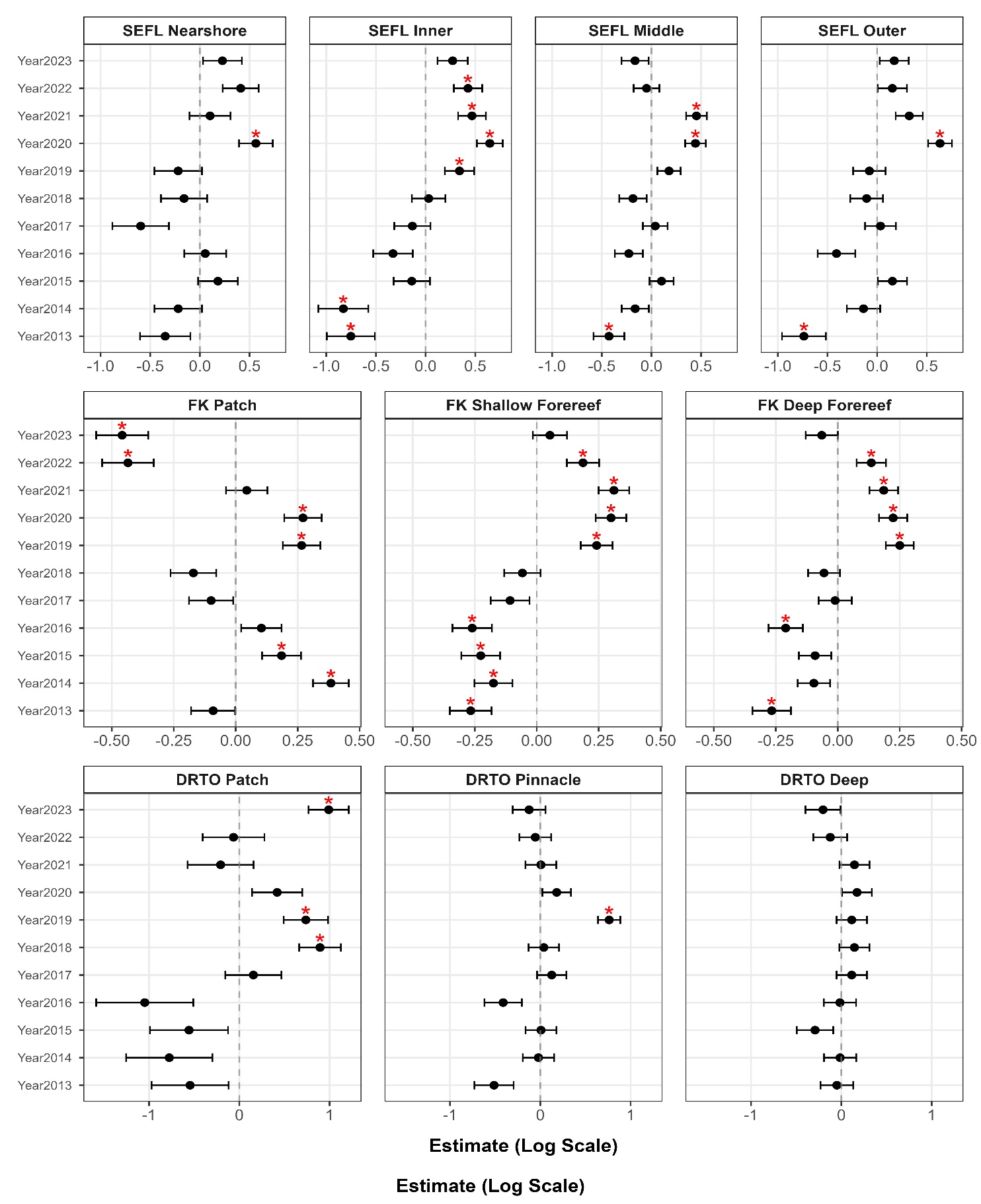


Figure S6. Yearly estimated marginal means (± SE) of target species recruit density (≤ 5 cm height) in contrast with regional habitat means from 2013 to 2023 in 10 regional habitats on Florida’s Coral Reef. Contrasts are based on the best-fit model and computed by the emmeans package in R. Regional habitats are organized by Southeast Florida (SEFL, top panel), Florida Keys (FK, middle panel), and Dry Tortugas (DRTO, bottom panel). In the FK and DRTO, target species included *Pseudoplexaura porosa, Antillogorgia bipinnata, Antillogorgia americana*, *Gorgonia ventalina,* and *Eunicea flexuosa*; only the last three species were targeted in SEFL. Depth increases left to right in each panel, but comparable depths are found between SEFL nearshore and inner reefs and between FK patch and shallow forereefs. Red asterisk indicates interannual significant differences.


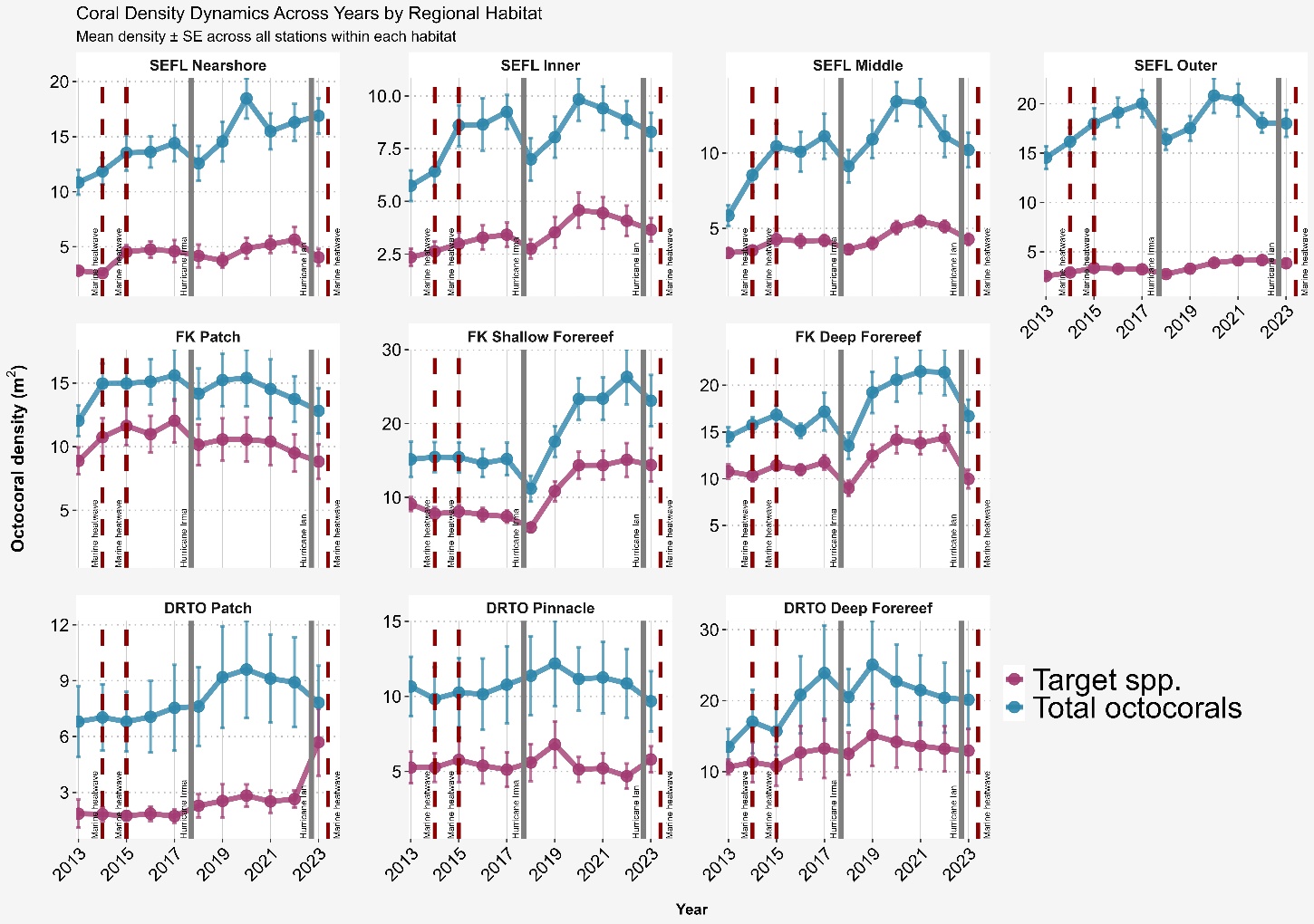


Figure S7. Arborescent octocoral mean (± SE) density for total octocorals (blue) and target species (purple) from 2013–2023 in 10 regional habitats on Florida's Coral Reef. The regional habitats are organized by Southeast Florida (SEFL, top panel), Florida Keys (FK, middle panel), and Dry Tortugas (DRTO, bottom panel). The depth increases left to right in each panel, but comparable depths are found between the SEFL nearshore and inner reefs and between the FK patch and shallow forereefs. Major disturbance events, including marine heatwaves and hurricanes, are indicated using vertical dashed and solid lines, respectively.
